# Single versus multiple hyperthermic intraperitoneal chemotherapy applications for T4 gastric cancer patients: Efficacy and safety profiles

**DOI:** 10.3389/fonc.2023.1109633

**Published:** 2023-03-17

**Authors:** Jing Zhang, Yuemin Sun, Xiaofeng Bai, Peng Wang, Liang Tian, Yantao Tian, Yuxin Zhong

**Affiliations:** ^1^ Department of Surgery, Huanxing Cancer Hospital, Beijing, China; ^2^ Department of Pancreatic and Gastric Surgery, National Cancer Center/National Clinical Research Center for Cancer/Cancer Hospital, Chinese Academy of Medical Sciences and Peking Union Medical College, Beijing, China

**Keywords:** lobaplatin, hyperthermic intraperitoneal chemotherapy, prognosis, metastasis, cancer

## Abstract

**Objective:**

To explore the clinical safety and efficacy of single and multiple applications of lobaplatin-based hyperthermic intraperitoneal chemotherapy (HIPEC) for patients with T4 gastric cancer and to evaluate the impact of HIPEC on peritoneal metastasis.

**Materials and methods:**

We retrospectively reviewed prospectively collected data from T4 gastric cancer patients who underwent radical gastric resection plus HIPEC between March 2018 and August 2020 from the National Cancer Center and Huangxing Cancer Hospital. Patients who underwent radical surgery and HIPEC were divided into two groups: the single-HIPEC group (radical resection + a single application of intraoperative HIPEC with lobaplatin 50 mg/m2 at 43.0 ± 0.5°C for 60 min), and a multi-HIPEC group (two more HIPEC applications were performed after radical surgery).

**Results:**

A total of 78 patients were enrolled in this two-center study; among them, 40 patients were in the single-HIPEC group, and 38 patients were in the multi-HIPEC group. The baseline characteristics were well balanced between the two groups. There was no significant difference in the postoperative complication rates between the two groups (P > 0.05). Mild renal dysfunction, mild liver dysfunction, low platelet levels and low white blood cell levels were recorded in both groups, without significant differences between the two groups (P > 0.05). After a mean follow-up of 36.8 months, 3 (7.5%) patients in the single-HIPEC group and 2 (5.2%) patients in the multi-HIPEC group experienced peritoneal recurrence (P > 0.05). Both groups had comparable 3-year overall survival (OS) (51.3% vs. 54.5%, P = 0.558) and 3-year disease-free survival (DFS) rates (44.1% vs. 45.7%, P = 0.975). Multivariate analysis showed that an age > 60 years and low preoperative albumin levels were independent risk factors for postoperative complications.

**Conclusion:**

Single and multiple applications of HIPEC in patients with T4 gastric cancer were safe and feasible. Both groups had similar postoperative complication rates, 3-year OS rates and 3-year DFS rates. Special attention should be given to HIPEC for patients aged > 60 years and patients with low preoperative albumin levels.

## 1 Introduction

Gastric cancer (GC) is one of the most common malignancies of the digestive tract (1). According to the 2020 Global Cancer Statistics Report, GC has the fourth highest morbidity rate and the second highest mortality rate worldwide (2). As early symptoms are usually atypical and inexpensive and noninvasive screening tools for diagnosis are lacking, patients are often diagnosed with advanced stage disease and thus have a poor prognosis (3). At present, the primary treatment for GC is radical surgical resection or its combination with radiotherapy, chemotherapy, and other treatment modes (4). Despite developments in treatment in recent years, patients with advanced GC generally have a worse prognosis, with a 5-year survival rate of less than 20% (5).

Peritoneal metastasis (PM) is the most common pattern of distant metastasis after radical surgery (6, 7). Currently, no curative treatment options exist for PM, and the outcome remains extremely poor; the 5-year survival rate is less than 3% (4). Hyperthermic intraperitoneal chemotherapy (HIPEC) is an effective means to reduce PM in patients with advanced GC (8, 9). HIPEC was initially applied to peritoneal carcinomatosis and involves raising the temperature of the local environment of the tumor cells, which results in induced cell death through apoptosis (10). In addition, hyperthermia is a sensitizing agent for chemotherapy, and both are beneficial to exterminating free tumor cells (11, 12). Cytoreductive surgery with HIPEC has been increasingly used in the treatment of multiple types of gastrointestinal surgeries; however, reports about the number of HIPEC applications remain limited (13, 14). To determine the safety and efficacy of radical surgery with HIPEC, especially the optimal number of HIPEC applications, for treating peritoneal metastasis and the postoperative complications, we retrospectively analyzed the clinical data of patients with T4 gastric cancer admitted to the Huangxing Cancer Hospital and National Cancer Center.

## 2 Materials and methods

### 2.1 Study population and grouping

We retrospectively collected data from 78 patients with T4 gastric cancer who underwent radical surgery with HIPEC from March 2018 to August 2020 at the Huangxin Cancer Hospital and National Cancer Center. This study protocol complied with the principles of the Declaration of Helsinki and was approved by the Institutional Review Board of the Cancer Hospital, National Cancer Center, Chinese Academy of Medical Sciences (NCC2017-YZ-026). After fully explaining of the benefits and disadvantages of HIPEC to patients and their families, patients chose whether to have single-HIPEC or multi-HIPEC, and informed consent forms were accordingly signed by all enrolled patients. The inclusion criteria were as follows: 1. histopathologically confirmed adenocarcinoma of the stomach; 2. pathological staging of T4, with or without lymph node metastasis; 3. no detected distant metastasis; and 4. at least 1 HIPEC applications. The exclusion criteria were as follows: 1. incomplete clinical data; 2. visceral metastasis was found during surgery, including peritoneal metastasis; 3. previous or coexistence of other malignant diseases; and 4. inability to complete the follow-up evaluation. Before surgery, hematological examination, contrast-enhanced computed tomography (CT), gastroscopy were routinely performed on all patients for tumor staging. And If lymph node metastasis was suspected based on preoperative imaging, neoadjuvant chemotherapy was performed. Finally, 78 patients with T4 gastric cancer met the inclusion criteria. Among them, 40 patients were included in the radical surgery plus single HIPEC application group (single-HIPEC), and 38 patients were included in the radical surgery plus multiple HIPEC applications group (multi-HIPEC).

### 2.2 Treatment method

All 78 patients received laparoscopic radical gastrectomy for gastric cancer (D2 lymphadenectomy), and the operations were performed by the same group of gastric surgeons. After general anesthesia and intubation were performed, the patients were placed in a supine position. First, a 1-cm long longitudinal incision was made below the navel as an observation hole, into which a 10 mm trocar was inserted. The other four trocars were placed in the left and right sides of the abdomen through operating holes. A pneumoperitoneum was established, and the pressure was maintained at 13 mmHg. Surgery started with exploration of the abdominal cavity; if there was no obvious sign of distant metastasis, laparoscopic-assisted radical gastrectomy was performed. According to the location of the tumor, total, distal subtotal, or proximal gastrectomy was performed. Following gastrectomy, Billroth-I, Billroth-II, or Roux-en-Y reconstruction was performed. Gastrectomy and digestive tract reconstruction were completed through an auxiliary incision (≤7 cm) above the navel. In the multi-HIPEC group, 4 abdominal drains were placed, and the drainage end was positioned in the left hepatorenal recess, subphrenic space and both sides of the pelvis. In the single-HIPEC group, two drainage tubes were routinely placed after the operation.

In the single HIPEC group, lobaplatin-based prophylactic HIPEC was performed under general anesthesia after closure of the incision. Lobaplatin (50 mg/m2) was diluted in a heated 5% glucose solution and then circulated for 60 min. The perfusion rate was 400-500 ml/min. The circulating temperature was maintained at 43.0 ± 0.5°C. After HIPEC, at least 90% of the perfusion fluid was removed. In the multi-HIPEC group, the second HIPEC application was started within 72 hours after surgery, and the third HIPEC application was applied 48 hours after the second. The second and the third application of HIPEC were the same as the first. The patients’ vital signs and drainage fluid color were observed carefully during HIPEC. All patients received adjuvant chemotherapy after radical surgical resection. Following the NCCN guidelines, the patients were followed up every three months for the first two years, every six months for the next three years, and then once a year after five years.

### 2.3 Follow-up

The first follow-up started 3 months after the end of the treatment cycles, and the follow-up was conducted using both outpatient and telephone appointments. The date of the last follow-up was March 20, 2022. The follow-up assessment comprised evaluations of postoperative complications, survival status, time of death, recurrence time, and tumor markers and CT imaging. The survival endpoints included disease-free survival (DFS) and overall survival (OS); DFS was defined as the time from surgery to tumor recurrence, while OS considered all deaths as events.

### 2.4 Statistical analysis

Statistical analysis was carried out using SPSS Statistics v24.0 software (IBM, Armonk, NY, USA) and GraphPad Prism (version 8, GraphPad Prism Software Inc.). The normally distributed measurement data are expressed as the mean ± SD, and a t test or analysis of variance was used for comparisons. Categorical data are shown as frequencies and percentages and were analyzed by the chi-squared test or Fisher’s exact test. Ranked and nonnormally distributed quantitative data were assessed by the Mann−Whitney test. Survival analysis was performed using Kaplan−Meier curves with the log-rank test. Differences were considered significant when the P value (*p*) was less than 0.05.

## 3 Results

### 3.1 Patient characteristics

In total, 78 patients met our inclusion and exclusion criteria. The patients had a mean age of 52.2 ± 9.3 years, and 55.1% were male. Of these patients, 40 patients were in the single-HIPEC group, and 38 patients were in the multi-HIPEC group. These two groups of patients were well balanced in terms of age, sex, ASA score, BMI, gastrectomy, tumor grade, pathological N staging, perineural invasion, tumor size, preoperative albumin level and preoperative hemoglobin level (**Table 1**).

**Table 1:** Baseline characteristics of the patients with T4 gastric cancer.

Characteristics	All patients	Single-HIPEC	Multi-HIPEC	*P value*
	(n = 78)	(n = 40)	(n = 38)	
Age at diagnosis, year, (n%)	52.2 ± 9.3	51.1 ± 9.5	53.3 ± 10.4	0.834
21-60	42 (54.8%)	21 (52.5%)	21 (55.3%)	
60-79	36 (46.2%)	19 (47.5%)	17 (45.7%)	
Sex, n (%)				0.633
Female	35 (44.9%)	16 (40.0%)	19 (50.0%)	
Male	43 (55.1%)	24 (60.0%)	19 (50.0%)	
ASA score				0.325
I	24 (30.8%)	12 (30%)	12 (31.6%)	
II	50 (64.1%)	26 (65.0%)	24 (63.2%)	
III	4 (5.1%)	2 (5%)	2 (5.3%)	
BMI, kg/m2 (mean ± SD)	24.3 ± 2.4	23.4 ± 3.4	25.4 ± 2.8	0.384
Gastrectomy, (n%)				0.693
Proximal	13 (16.7%)	7 (17.5%)	6 (15.8%)	
Distal	51 (65.4%)	27 (67.5%)	24 (63.2%)	
Total	14 (17.9%)	6 (15.0%)	8 (21.0%)	
Tumor grade, (n%)				0.784
Poor or moderate	51 (65.4%)	29 (72.5%)	22 (57.9%)	
Mucinous or signet cell	27 (34.6%)	11 (27.5%)	21 (42.1%)	
Pathological N staging, (n%)				0.424
N0	7 (9.0%)	4 (10.0%)	3 (7.9%)	
N1	28 (35.9%)	15 (37.5%)	13 (34.2%)	
N2	37 (47.4%)	20 (50.0%)	17 (44.7%)	
N3	6 (7.7%)	1 (2.5%)	5 (13.2%)	
Perineural invasion, (n%)				0.553
Yes	55 (70.5%)	25 (62.5%)	30 (78.9%)	
No	23(29.5%)	15 (37.5%)	8 (21.1%)	
Neoadjuvant CHT				0.428
Yes	56 (71.8%)	27 (67.5%%)	29 (76.3%)	
No	22 (28.2%)	13 (32.5%)	9 (23.7%)	
Tumor size, cm, (n%)				0.214
0-5	40 (51.3%)	24 (60.0%)	16 (42.1%)	
> 5	38 (48.7%)	16 (40.0%)	22 (57.9%)	
Preoperative albumin level, g/L	36.3 ± 3.4	36.5± 3.3	35.8± 3.6	0.829
Preoperative hemoglobin level, g/L	109.4 ± 8.4	108.3 ± 9.6	109.6 ± 8.5	0.733

Conversion to open surgery was reported in 3 (7.5%) patients in the single-HIPEC group and 2 (5.3%) in the multi-HIPEC group (p = 0.885). The mean operation time in the single-HIPEC group was 203 ± 32.8 mins, compared with 186 ± 36.2 mins in the multi-HIPEC group, and no significant difference was observed (p = 0.122). There were no significant differences in estimated blood loss, hospital stay after the operations and postoperative gastrointestinal recovery. Two patients in the single-HIPEC group and one patient in the multi-HIPEC group suffered from anastomotic leakages (p = 0.871). In each group, 3 patients suffered from wound healing problems. Five (12.5%) patients in the single-HIPEC group 5 (13%) patients in the multi-HIPEC group had abnormal liver function (elevated ALT level) (p = 0.068). All patients recovered to have normal liver function after being given hepatoprotective drugs. Seven (17.5%) patients in the single-HIPEC group demonstrated a decline in their peripheral platelet count, including 6 (15.6%) patients in the multi-HIPEC group (p = 0.644); however, none of the patients in either group experienced major bleeding events leading to anemia. There were no cases of 30-day postoperative mortality in either group. No severe neurotoxicity was observed in either group. During the follow-up period, 3 (5.5%) patients in the single-HIPEC group and 2 (5.2%) patients in the multi-HIPEC group suffered from peritoneal recurrence (**Table 2**).

**Table 2:** Perioperative data of the patients.

Characteristics	Single-HIPEC	Multi-HIPEC	*P value*
	(n = 40)	(n = 38)	
Conversion to open, n (%)	3 (7.5%)	2 (5.3%)	0.885
Operation time in min, mean ± SD	203 ± 32.8	186 ± 36.2	0.122
Estimated blood loss in mL, mean ± SD	80.3 ± 22.6	72.4 ± 27.8	0.358
Postoperative hospital stay (d, mean ± SD)	7.0 ± 1.4	7.3 ± 0.9	0.486
Thirty-day postoperative mortality, n (%)	0	0	1
Time to first flatus, day (mean±SD)	2.4 ± 1.5	2.3 ± 1.4	0.682
Time to regular diet, day (mean±SD)	5.3 ± 2.3	5.4 ± 2.7	0.655
Postoperative complications (grades III, IV)			
Intra-abdominal infection	0	0	1
Pleural effusion	1(2.5%)	0	0.757
Anastomotic leakage	2 (5.0%)	1 (2.6%)	0.732
Bowel obstruction	2 (5.0%)	2 (5.2%)	0.871
Surgical wound infection	3 (7.5%)	3 (7.8%)	0.855
Bleeding	1 (2.5%)	1 (2.6%)	0.935
Delayed gastric emptying	5 (12.5%)	6 (15.7%)	0.557
Lung infection	1 (2.5%)	0	0.866
Fever	4 (10.0%)	3 (7.8%)	0.342
Abnormal blood routine tests	7 (17.5%)	6 (15.6%)	0.644
Abnormal renal function	1 (2.5%)	1 (2.6%)	0.956
Abnormal liver function	5 (12.5%)	5 (13%)	0.068
Severe neurotoxicity	0	0	1
Peritoneal recurrence	3 (5.5%)	2 (5.2%)	0.379

We performed multivariable logistic regression analysis to identify factors influencing postoperative complications. The results showed that an age older than 60 years and low preoperative albumin levels were significantly associated with postoperative complications (**Table 3**).

**Table 3:** Multivariable analysis of factors influencing postoperative complications.

Characteristics	OR (95% CI)	*P* value
Age at diagnosis, year		
21-60	1 [Reference]	
60-79	2.133 (1.734-2.898)	0.036
Sex		
Female	1 [Reference]	
Male	1.116 (0.866-1.327)	0.552
ASA score		
I	1 [Reference]	
II	0.744 (0.557-1.382)	0.433
III	1.240 (0.833-1.562)	0.382
Gastrectomy		
Proximal	1 [Reference]	
Distal	0.569 (0.381-1.146)	0.072
Total	0.769 (0.626-1.115)	0.238
Tumor grade		
Poor or moderate	1 [Reference]	
Mucinous or signet cell	1.234 (0.988-1.427)	0.088
Pathological N staging		
N0	1 [Reference]	
N1/N2/N3	1.247 (1.044-1.354)	0.115
Treatment modality		
Surgery plus single-HIPEC	1 [Reference]	
Surgery plus multi-HIPEC	0.896 (0.732-1.324)	0.349
Preoperative albumin level		
Normal or above	1 [Reference]	
Below normal	1.756 (1.532-2.194)	0.031
Preoperative hemoglobin level		
Normal or above	1 [Reference]	
Below normal	0.882 (0.643-1.218)	0.263

### 3.2 Survival analysis

**Figure 1:**
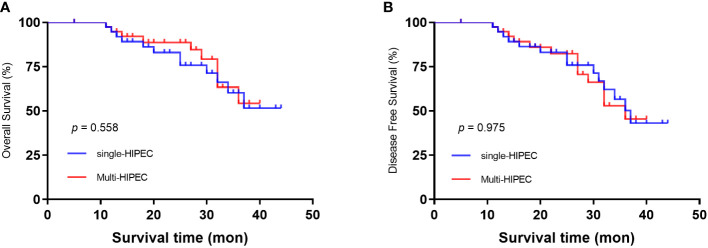
Kaplan‒Meier survival curves depicting the survival in patients with T4 gastric cancer. **(A)** Patient OS based on whether single or multi-HIPEC was performed. **(B)** Patient DFS based on whether single or multi-HIPEC was performed.

The mean follow-up time was 36.8 months. All patients also underwent postoperative adjuvant chemotherapy (oxaliplatin plus capecitabine for 6 cycles). The two groups had comparable 3-year overall survival rates (51.3% single-HIPEC group vs. 54.5% multi-HIPEC group, P = 0.558) (**Figure 1A**) and 3-year disease-free survival rates (44.1% versus 45.7%, p = 0.975) (**Figure 1B**).

## 4 Discussion

Radical surgery with HIPEC has been proven to be an effective treatment modality for selected patients with peritoneal malignancies (15–17). In recent years, the safety of using HIPEC as an adjuvant treatment for advanced gastric cancer has been reported in several randomized clinical trials; however, there is still a lack of investigations on the difference between single and multiple applications of HIPEC and their effects on peritoneal recurrence (18–20).

This is a two-center, retrospective study, and the purpose of this study was to investigate the safety and efficacy of single and multiple applications of HIPEC for patients with T4 gastric cancer and to evaluate their impact on peritoneal metastasis. The results showed that single and multiple applications of HIPEC in patients with T4 gastric cancer were both safe and feasible. The two groups had similar postoperative complication rates, 3-year OS rates and 3-year DFS rates. The results also indicated that special attention should be given to HIPEC patients aged over 60 years and patients with low preoperative albumin levels.

In approximately 30% ~ 50% of patients with T4 gastric cancer, intraperitoneal free cancer cells can be detected during surgery (21), and the stress of anesthesia and surgery results in immune suppression, rendering the free cancer cells from the surveillance of the immune system, which ultimately causes peritoneal recurrence. Lee et al. reported that the rate of peritoneal metastasis after D2 resection of advanced gastric cancer was as high as 58.8%, and the median survival duration in these patients was shorter than that in patients with other types of distant metastasis (22). Peritoneal metastasis is the principal cause of mortality in patients with advanced gastric cancer (23, 24). Beeharry et al. conducted a study that included 80 patients with gastric cancer and divided them into a radical surgery plus HIPEC group (40 patients) and a surgery alone group. Their results showed that compared with the surgery alone group, patients who received HIPEC had better DFS and peritoneal recurrence rates. However, due to the small sample size of the two groups, the rate of perioperative adverse events was not compared between the two groups (25). The results of our study indicate that the number of HIPEC applications had no statistical effect on 3-year OS or 3-year DFS.

HIPEC technology has been continuously developed and improved over time, and HIPEC-related complication rates and fatality rates have gradually decreased (26, 27). Jafari et al. performed a retrospective study of 694 patients who received HIPEC after surgery and evaluated the efficacy and safety of this treatment. The results demonstrated that postoperative bleeding, pulmonary infection, respiratory failure and septic shock were the most predominant complications after HIPEC, but there was no significant difference between the two groups in anastomotic leakage rate, intestinal adhesion rate or intestinal obstruction rate (28). Jennifer et al. also showed that there was no significant difference in gastrointestinal-related complications between the HIPEC group and the control group (29). In our study, our results also showed that the incidence of postoperative complications, such as anastomotic leakage, intestinal obstruction, wound infections, and postoperative bleeding, in patients with T4 gastric cancer treated with HIPEC after surgery was acceptable. Therefore, properly increasing the number of HIPEC procedures after surgery will not increase the risk of adverse events in these patients.

The advantages of HIPEC include minimizing the adverse effects common with systemic chemotherapy, increasing the drug sensitivity of tumor cells and prolonging the exposure of the drug to tumor cells, all of which lead to increased tumor killing with fewer adverse effects (4, 15, 23). To further evaluate the possible factors that may influence postoperative complications, we conducted a multivariate logistic regression analysis. The results demonstrated that an age older than 60 years and low preoperative albumin levels were significantly associated with postoperative complications, and the number of HIPEC applications showed no correlation with the occurrence of postoperative complications. Body functions and the ability to repair surgical trauma weaken with age. Therefore, it is predictable that advanced age is an independent risk factor for postoperative complications (30). Also, several studies have similar conclusion, low preoperative albumin level plays an important role in judging the prognosis of patients with gastric cancer and can provide information to guide clinical practice (31, 32). In the future, we would like to futher explore the underlying mechanism between preopetative albumin level and prognosis in GC cancer patients. In 2017, Desiderio et al (20) published a meta-analysis that included 32 trials (2520 patients). Their results showed that the 3-year OS rate and 5-year OS rate in the HIPEC group were significantly higher than those in the non-HIPEC group, and HIPEC led to a great opportunity to improve the outcomes of patients with peritoneal metastasis. However, in our study, we found that the number of HIPEC applications did not significantly affect 3-year OS (p = 0.558) or 3-year DFS (p = 0.975).

This study has several limitations. First, this was a two-center study, and the number of enrolled patients was relatively small, which may impact the credibility of the results. Second, the follow-up durations were relatively short, and it is possible that the follow-up period was not long enough to observe differences between these two groups. Third, the present study was a retrospective study, and selection bias may have been present. Prospective multicenter studies with large samples and long-term follow-up are required to confirm our results.

In conclusion, our study shows that both single and multiple HIPEC applications are feasible and safe for patients with T4 gastric cancer and have similar postoperative complication rates, 3-year OS rates and 3-year DFS rates. Special attention should be given to HIPEC in patients aged over 60 years and patients with low perioperative albumin levels.

## Data availability statement

The raw data supporting the conclusions of this article will be made available by the authors, without undue reservation.

## Author contributions

JZ and YS: acquisition of data. JZ: analysis and interpretation of data. XB, PW and LT drafted the manuscript. YT and YZ: critical revision of the manuscript. All authors contributed to the article and approved the submitted version. 
